# Analysis of progress and challenges for various patterns of c-MET-targeted molecular imaging: a systematic review

**DOI:** 10.1186/s13550-017-0286-z

**Published:** 2017-05-08

**Authors:** Zhaoguo Han, Yongyi Wu, Kai Wang, Yadi Xiao, Zhen Cheng, Xilin Sun, Baozhong Shen

**Affiliations:** 1 Molecular Imaging Research Center, Harbin Medical University, 766Xiangan N street, Songbei District, Harbin, Heilongjiang 150028 China; 2 TOF-PET/CT/MR center, The Fourth Hospital of Harbin Medical University, Harbin, Heilongjiang China; 3 Molecular Imaging Program at Stanford (MIPS), Department of Radiology, Stanford University School of Medicine, Lucas Center, Room P089, 1201 Welch Rd, Stanford, CA 94305-5484 USA

**Keywords:** Molecular imaging, Malignancy, c-MET, Targeted molecular probe, Effectiveness evaluation

## Abstract

**Background:**

Mesenchymal–epithelial transition factor also named c-MET is a receptor tyrosine kinase for the hepatocyte growth factor that plays a pivotal role in tumorigenesis. c-MET-targeted therapies have been tested in preclinical models and patients, with significant benefits for cancer treatment. In recent years, many studies have shown that the expression level and activation status of c-MET are closely correlated to c-MET-targeted therapy response and clinical prognosis, thus highlighting the importance of evaluating the c-MET status during and prior to targeted therapy. Molecular imaging allows the monitoring of abnormal alterations of c-MET in real time and in vivo.

**Results:**

In this review, we initially summarize the recent advances in c-MET-targeted molecular imaging, with a special focus on the development of imaging agents ranging in size from monoclonal antibody to small molecule. The aim of this review is to report the preclinical results and clinical application of all molecular imaging studies completed until now for in vivo detection of c-MET in cancer, in order to be beneficial to development of molecular probe and the combination of molecular imaging technologies for in vivo evaluation of c-MET. Various molecular probe targeted to c-MET possesses distinctive advantages and disadvantages. For example, antibody-based probes have high binding affinity but with long metabolic cycle as well as remarkable immunogenicity.

**Conclusions:**

Although studies for c-MET-targeted molecular imaging have made many important advances, most of imaging agents specifically target to extracellular area of c-MET receptor; however, it is difficult to reflect entirely activation of c-MET. Therefore, small molecule probes based on tyrosine kinase inhibitors, which could target to intracellular area of c-MET without any immunogenicity, should be paid more attention.

## Background

### Clinical relevance of the HGF/c-MET signaling pathway: structure, function, and dysregulation

Mesenchymal–epithelial transition factor (c-MET) is a receptor tyrosine kinase (RTK) naturally activated by the binding of hepatocyte growth factor (HGF), and then regulates many essential cellular processes including cell proliferation, motility, invasion, angiogenesis, and apoptosis. The c-MET oncogene is located on chromosome 7q21-31 and was initially identified in an immortalized osteosarcoma cell line by Cooper et al. in the1980s [1]. The c-MET receptor is located in the cell surface and is expressed in epithelial cells of many organs including the liver during both embryogenesis and adulthood [2].

The mature form of the c-MET receptor is a disulfide-linked heterodimer consisting of a 50-kDa extracellular α-chain and a transmembrane 140-kDa β-chain. Both the α chain and the first 212 residues of the β chain constitute the Sema domain, which has an HGF-binding site in the extracellular region [3].

HGF [4, 5], also known as scatter factor [6], is the only endogenous ligand for c-MET [7]. HGF is a 90-kD multidomain glycoprotein that is similar to plasminogen, a circulating proenzyme. HGF is cleaved by proteases to form, similarly to its receptor, α/β heterodimers linked by a disulfide bond, thereby converting it into the active form [8]. The NH_2_-terminal portion of the HGF α chain contains the high affinity c-MET binding domain, and the β chain is responsible for activating c-MET receptor via a direct interaction.

After HGF-binding, the c-MET receptor undergoes homodimerization and phosphorylation at tyrosines 1234 and 1235 and subsequently at tyrosines 1349 and 1356 in the carboxy-terminal tail [9]. After phosphorylation, these tyrosines recruit GRB2 (adaptor protein growth factor receptor bound protein 2) [10], SHC (Srchomology-2-containing) [11] and CRK (v-crk sarcoma virus CT10 oncogene homolog), CRKL [12, 13], PI3K (the effector molecule phosphatidylinositol 3-kinase), PLCγ (phospholipase Cγ), and a series of signaling effectors that have been described in detail in previous reviews [14–17]. Similarly to many RTKs, components of the c-MET downstream signaling pathways such as MAPK will translocate into the nucleus and bind transcription factors to regulate the transcription of specific genes involved in a variety of cellular processes including cell proliferation, motility, and cell cycle progression [10, 18]. Other major downstream axes of c-MET signaling include the PI3K/Akt and STAT/JNK signaling axes, responsible for cell survival and transformation, respectively [13, 19].

It is known that dysregulation of many RTKs, including c-MET, contributes to tumorigenesis [20]. Indeed, c-MET is activated in a variety of cancers, such as renal, ovarian, and lung. [8, 21]. Aberrant activation of c-MET results in proliferation, invasion, angiogenesis, and inhibition of apoptosis [19, 22, 23]. Conversely, downregulation of MET expression in tumor cells can lower their tumorigenic potential [24]. Studies indicated that c-MET in cancer is activated through both ligand-dependent autocrine or paracrine mechanisms and ligand-independent mechanisms including gene amplification, gene translocation, activating mutations, or transcriptional upregulation of the c-MET protein [25, 26]. Furthermore, in some studies, a synergistic effect for phosphorylation between c-MET and epidermal growth factor receptor (EGFR), HER2, HER3, RAS, RON, or PDGFR was found in some cancer cells or transfected cancer cells, and these studies might have important significance for combination therapy in many cancers [27–32].

c-MET is an attractive drug target in cancer therapy, and various targeted drugs have been used in multiple clinical trials as cancer therapeutic agents [33]. Furthermore, combined therapy targeted to c-MET and EGFR (onartuzumab + erlotinib) has significantly reduced resistance to treatment in preclinical studies when compared to erlotinib alone, but failed to get consistent result in a clinical trial [34–36]. Maybe more comprehensive clinical setting needs to be performed to confirm the prospective viewpoint.

### Molecular imaging of c-MET

As c-MET is an active participant in tumorigenesis and the malignant progress, the assessment of c-MET activation status in real time could be valuable for diagnosis and monitoring of responses to targeted therapies in cancer in the future. While c-MET detection by immunohistochemistry or fluorescent in situ hybridization (FISH) is currently the standard diagnostic procedure, these methods require repeated biopsies that can be painful for the patient. Thus, the development of more effective noninvasive detection methods for accessing c-MET expression and activation is needed.

**Fig. 1:**
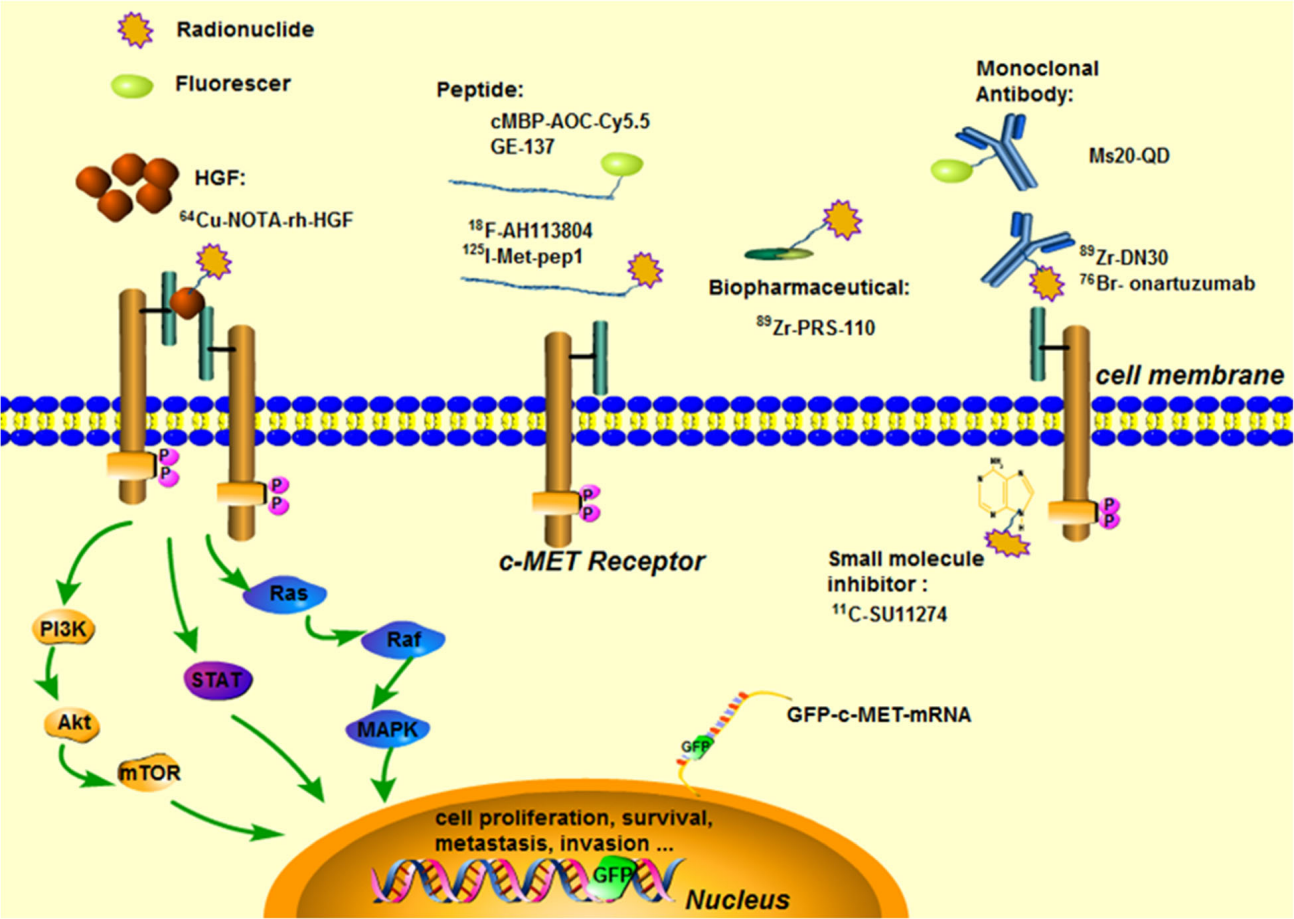
A visual overview of the c-MET/HGF signaling pathway with different biological effects and also of representative imaging agents targeting to either extracellular or intracellular domains of c-MET receptor

Molecular imaging is a noninvasive method that can provide accurate information in vivo and in real time, and its application for detecting c-MET activation could represent a breakthrough in cancer diagnosis. Various c-MET-targeted tracers have been developed based on multiple molecular modalities, such as antibody, peptide, small protein, genetically encoded protein, and small molecule tyrosine kinase inhibitor (TKI). These tracers also have been further evaluated in preclinical studies or clinical setting. Recent studies acquired significant progress toward the development of molecular imaging probes for monitoring c-MET activation, but the imaging performance of these probes remains a critical issue. Here, we review and discuss these studies (Fig. 1, Table 1).

**Table 1:** Representative molecular imaging agents developed for c-MET imaging in cancer

Name	Nature	Molecular weight (kDa)	Targeted area	Tumor model	Tumor uptake	T/C	Imaging technique	Time point	Clin
^64^Cu-NOTA-rh-HGF	rh-ligand	~90	Extracellular	U87MG	6.8 ± 1.8%ID/g	T/M =13.5 ± 6.1	PET	9 h p.i.	No
^125^I-mAb	mAb	~150	Extracellular	S114	–	T/TB = 0.37	Gamma camera	3 days p.i.	No
^125^I-Met3	mAb	~150	Extracellular	S114, SK-LMS-1/HGF	18.6 ± 2.1%EIA, 7.2 ± 2.2%EIA	T/TB = 0.32 ± 0.13, T/TB = 0.168 ± 0.076	Gamma camera	3 days p.i., 6 days p.i.	No
^125^I-Met5	mAb	~150	Extracellular	SK-LMS-1/HGF	13.3 ± 7.6%EIA,	T/TB = 0.146 ± 0.097	Gamma camera	1 day p.i.	No
^125^I-hFab-Met-1	Fab fragment	~60	Extracellular	SK-LMS-1/HGF	6%EIA	–	Gamma camera	5 h p.i.	No
^125^I-MET4	mAb	~150	Extracellular	SK-LMS-1/HGF	–	–	Gamma camera	3 days p.i.	No
^89^Zr-DN30	mAb	~150	Extracellular	GTL-16	19.6 ± 3.3%ID/g	T/M = 17.5	PET	3 days p.i	No
^89^Zr-df-onartuzumab	mAb	~99	Extracellular	MKN-45	23%ID/g	T/M = 27	PET	3 days p.i	No
^89^Zr-DFO-H2cys-diabody	scFv	~55	Extracellular	Hcc827-GR6	3.4 ± 0.3%ID/g	T/M =27 ± 14	PET	44 h p.i.	No
Ms20-QD	scFv	~25	Extracellular	H1993	–	T/T_n_ = 4.3	NIRfluorescence	24 h p.i.	No
anti-MET-Gd-DTPA-albumin	mAb	~150	Extracellular	C6	–	SIC(T/T_n_) ≈ 5	MRI	3 h p.i.	No
SPIO-anti-c-MET	PcAb	~150	Extracellular	CDAA-HCC	–	SIC(T/T_n_) ≈ 2	MRI	1.5 h p.i.	No
^125^I-Met-pep1	Peptide	~1.5	Extracellular	SK-LMS-1/HGF	5%EIA,	–	Gamma camera	4 h p.i.	No
^125^I-cMBP-GGG	Peptide	~1.9	Extracellular	U87MG	7.05 ± 1.2%ID/g	T/Bl = 2.94	SPECT	4 h p.i.	No
cMBP-AOC-Cy5.5	Peptide	~2.8	Extracellular	U87MG	–	T/M =33.711 ± 3.34	NIRfluorescence	5 h p.i.	No
GE-137	Peptide	~4.2	Extracellular	SKOv3	–	T/Ba = 1.6	NIRfluorescence	3 h p.i.	Yes
^18^F-AH113804	Peptide	~3.2	Extracellular	HCC1954	1.5 ± 0.2%ID/ml	T/M ≈ 2	PET	1 h p.i.	No
^99m^Tc-AH113018	Peptide	~3.2	Extracellular	MKN-45	10.1 ± 1.5%ID/g	T/M =55.2 ± 13.4	SPECT	1.5 h p.i.	No
^89^Zr-PRS-110	s-protein	57	Extracellular	H441	7.5 ± 3.4%ID/g	T:M = 29	PET	48 h p.i.	No
^11^C- SU11274	smTKI	~0.6	Intracellular	H1975	SUV = 0.45	T/T_N_ = 2.6	PET	1.3 h p.i.	No

*Tumor model* major positive tumor model, *Tumor uptake* highest tumor uptake in major positive tumor model, *T*/*C* tumor control area activity ratio, *Time point* optimal imaging time point, *Clin* clinical transformation, %*EIA* percentage of estimated injected activity, *SUV* standard uptake value, *T*/*M* tumor to muscle, *T*/*TB* tumor to total body, *T*/*T*_*n*_ tumor to tumor with non-specific probe, *SIC* signal intensity changes, *T*/*Bl* tumor to blood, *T*/*Ba* tumor to background fluorescence, *T*/*T*_*N*_ tumor to negative tumor

## Review

### Various patterns of molecular probes targeted to c-MET

#### Molecular imaging agents based on the HGF ligand

It is well established that HGF can alter the hemodynamics and metabolism of normal and malignant c-MET-expressing tissues [37, 38], and it has been gradually recognized that HGF has high binding affinity and specificity to c-MET. Initial studies on targeted molecular imaging of c-MET were therefore mainly based on HGF ligands. Shaharabany et al. [39] demonstrated in 2001 that hemodynamic changes induced by HGF correlated to c-MET activation in vivo. HGF was injected into xenografts in mice model, and then the mice were imaged by magnetic resonance imaging (MRI) and Doppler ultrasound. Results measured by blood oxygenation level dependent (BOLD)-MRI indicated that organs and tumors expressing c-MET levels showed more substantial alteration in blood oxygenation levels than those without c-MET expression. For instance, 60% of MRI signal alteration was detected in the liver, which has high levels of c-MET expression, whereas in the kidneys, only 30% signal alteration was detected, and no change was observed in the muscles. In mice bearing tumors derived from DA3 (murine mammary adenocarcinoma) cell line expressing high levels of c-MET, significantly higher signal intensity was detected, and in particular within the tumors when compared to the tumor periphery. The hemodynamic impact in the liver and kidneys in these mice were similar to those measured in the mice bearing tumors with low c-MET expression described above. In this elegant study, the authors show that MRI signal alterations are not only specific to c-MET activation but they are also dose dependent, as injection of HGF at 7 and 170 ng/g body weight resulted in signal alterations of 5 and 30% respectively. Similarly, in 2006, Tsarfaty et al. imaged c-MET activation in vivo indirectly by microbubble contrast medium (CM) ultrasound imaging after administration of HGF in murine models. Interestingly, the “newly opened” vessels (vessels that could not be detected before administration of HGF) caused by HGF treatment were mainly responsible for tumor blood volume increase [40].

**Fig. 2:**
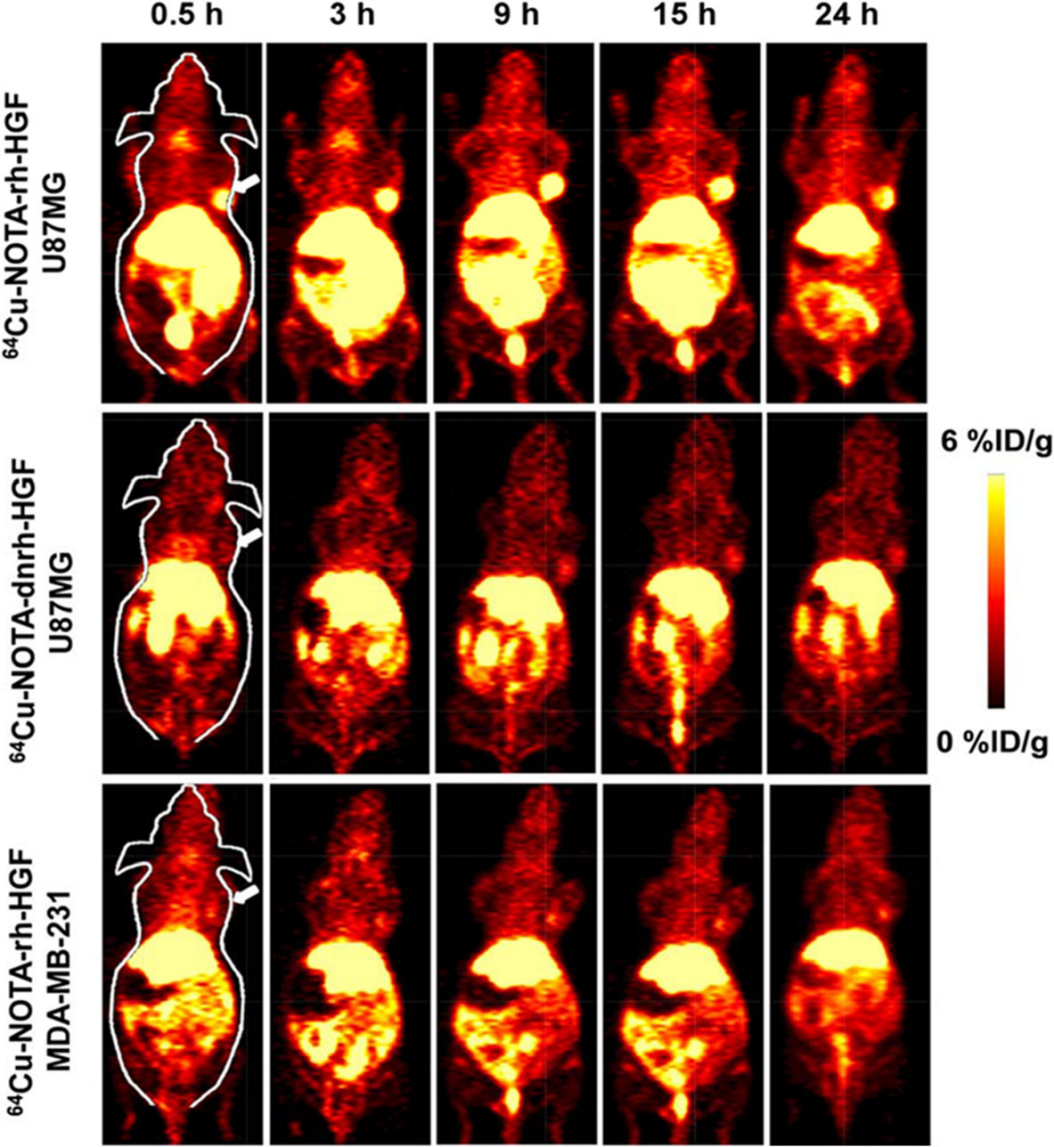
Coronal micro-PET images of c-Met expression in U87-MG and MDA-MB-231 bearing mice at serial timepoint post-injection of ^64^Cu-NOTArh-HGF or ^64^Cu-NOTA-dnrh-HGF are shown, and tumors are indicated by *arrows*. With permission from reference [41]

More recently, in 2015, Luo et al. reported the synthesis of ^64^Cu-NOTA-rh-HGF (recombinant human hepatocyte growth factor) and evaluated its potential as a PET imaging radiotracer for c-MET-targeted molecular imaging [41]. The synthesis of ^64^Cu-NOTA-rh-HGF was accomplished by conjugating concentrated rh-HGF to p-SCN-Bn-NOTA and subsequently chelating with ^64^Cu. Flow cytometry examination in U87-MG (human glioblastoma) and MDA-MB-231(human breast cancer) cell lines, which have moderate level and low level expression of c-MET, respectively, confirmed the specific binding capacity of rh-HGF to c-MET. In vivo studies further revealed that tumor uptake of ^64^Cu-NOTA-rh-HGF was rapidly and clearly visible at 0.5 h post-injection (p.i.) and peaked at 9 h p.i. (6.7 ± 1.8%ID/g) in U87-MG xenografts in mice; however, it was significantly lower in MDA-MB-231 xenografts in mice (1.8 ± 0.6%ID/g at 9 h p.i.), with consistency between PET images and biodistribution data in both models (Fig. 2). On the other hand, ^64^Cu-NOTA-dnrh-HGF, which was heat denatured and hence could not bind to c-MET, had significantly lower uptake in U87-MG xenografts than ^64^Cu-NOTA-rh-HGF, with the highest value at 1.6 ± 0.6%ID/g at 15 h p.i. Furthermore, probe uptake in all major organs was comparable between ^64^Cu-NOTA-rh-HGF and ^64^Cu-NOTA-dnrh-HGF, further confirming the specificity of the tracer to c-MET. The liver and kidney uptake of ^64^Cu-NOTA-dnrh-HGF were comparable to those of mice injected with ^64^Cu-NOTA-rh-HGF, as expected since these should be the clearance organs for a tracer of this size. With a molecular weight of ~90 kDa, rh-HGF is significantly smaller than monoclonal antibodies (mAbs) (typically 150 kDa). Therefore, the smaller ^64^Cu-NOTA-rh-HGF displayed a faster circulation clearance than radiolabeled intact mAbs, which also obtained a higher optimal tumor/muscle ratio (13.5 ± 6.1 at 9 h p.i. in U87-MG xenografts) in less than a half-life period.

These studies demonstrate that imaging agents based on the endogenous HGF ligand could be a straightforward and effective approach to image c-MET. However, while HGF displays good pharmacokinetic properties and high selectivity to c-MET, it also presents significant disadvantages. For instance, endogenous ligands will inevitably induce biological effects such as increased cell proliferation and enhanced cell survival and could also stimulate tumor growth. In addition, the endogenous ligand will theoretically preclude the preferential uptake of the radiolabeled ligand versus the unlabeled ligand in animal tumor models and therefore reduce contrast ratio of the tumor PET or SPECT images [42]. These limitations hinder the clinical translation of HGF-based molecular imaging and need be addressed in the future.

#### Molecular imaging agents based on antibodies

In the past two decades, several monoclonal antibodies (mAbs) against the HGF/c-MET signaling pathway including rilotumumab, ficlatuzumab, DN30, and onartuzumab, among others, have been extensively studied in preclinical and clinical trials, with promising outcomes in cancer treatment. Consequently, antibodies have also become of interest in the search for viable imaging agents for c-MET-targeted molecular imaging.

Preliminary work by Hay et al. tested a mAbs-based radiotracer mixture for molecular imaging of c-MET [43]. The radioiodinated mAb mixture (^125^I-mAb) consisted of anti-c-MET mAb and anti-HGF mAb. So they can target tumor cells directly and indirectly by binding to c-MET and HGF, respectively. The ^125^I-mAb mixture was synthesized with a radiolabeling efficiency of >60 and >85%, and the radioactivity was detected by whole body gamma camera imaging as early as 1 h p.i. in SK-LMS-1 (human leiomyosarcoma with high expression of c-MET) and S-114 (NIH 3T3 cells transformed with human HGF and human c-MET) murine models, whereas initial visualization of tumors in M-114 and DA3 murine models were at 1 day p.i. (M-114, NIH 3 T3 cells transformed with murine HGF and murine c-MET; DA3, mouse mammary carcinoma expressing murine c-MET). Quantitative ROI (region of interest) analyses indicated that tumors with autocrine for human HGF and c-MET demonstrated more rapid uptake and clearance of the ^125^I-mAb mixture than tumors expressing one or both murine homologues, reaching a mean value of tumor-to-whole body activity ratio by >0.3 at 1 day p.i. Generally, mice bearing “human” tumors (SK-LMS-1 and S-114) display a faster uptake and a faster metabolism of tracer from body than mice bearing “murine” tumors (M-114, DA3), as shown in images (lower levels of splanchnic activity at 3 and 5 days p.i. and more conspicuous thyroid activity derived from uptake of free radioiodide released by mAb deiodination). This study did not however include a HGF/c-MET negative control group. In a follow-up study, Hay et al. compared two anti-c-MET mAbs, Met3 and Met5, for nuclear imaging of human and canine c-MET-expressing tumor xenografts in nude mice [44]. Met5 and Met3 were radioiodinated with ^125^I by the same procedure described previously, with a radiolabeling efficiency of >60 and >90% [43]. In qualitative terms, Met3 and Met5 had comparable results of PC-3 tumors (human prostate cancer with high expression of c-MET) and SK-LMS-1/HGF tumors in mice models with high tumor/whole body ratio. In quantitative ROI analyses, both mice models injected with ^125^I-Met3 exhibited the highest uptake value at the earliest imaging time point (typically 1 to 2 h post-injection) and declined over time, similarly to previous research [45]. However, ^125^I-Met5-injected xenografts in mice models exhibited the highest uptake value at 1 day p.i., with respectively 13.3 ± 7.6%EIA (percentage of estimated injected activity) in SK-LMS-1/HGF, 7.5 ± 3.4%EIA in PC-3, and remained high until the final imaging point (5–6 days after injection). Although quantitative analysis indicated faster tumor uptake for ^125^I-Met3, ^125^I-Met5 obtained higher and more durable tumor uptake (at least 5 days). Therefore, Met5 might be more useful for radioimmunotherapy. Inevitably, distinct thyroid activity was observed since 1 day p.i. caused by deiodination from tracers. For ^125^I-LF7, a negative control mAb, the tumor uptake was <3% EIA at any time point. Ex vivo tissue uptake measurements were however not performed in this study.

To reduce the effects of c-MET activation induced by mAbs or native ligands on c-MET expression in vivo, Jiao et al. assessed the feasibility of using a monovalent anti-c-MET antigen-binding fragment (Fab, ~60 kDa) as a molecular probe for targeted imaging of c-MET [46]. hFab-Met-1 was selected from a large human naive Fab phage-display library with high specificity to c-MET but without agonist activity to HGF/c-MET signaling, as confirmed by in vitro assays. In an in vivo study, activity started to accumulate in tumor regions at 1–2 h after injection of ^125^I-hFab-Met-1, it was highly accumulated at 5–8 h p.i. and persisted for at least 24 h in SK-LMS-1/HGF bearing mice models, with a mean tumor activity of about 6% of the estimated activity administrated. Particularly high accumulation of activity was detected in the thyroid region. Although possessing smaller molecular weight and therefore faster metabolism as well as tumor uptake than the whole antibody, the Fab single fragment has a lower binding affinity than the whole antibody, as we have seen for ^125^I-hFab-Met-1, obtained highest tumor uptake of 6%EIA which is lower than ^125^I-Met5 (~13%EIA).

In 2009, Knudsen et al. [47] developed a new mAb (MET4) to use as a radiotracer for c-MET-targeted molecular imaging. MET4 targets overexpressed c-MET in tumors by binding to a specific epitope located in the extracellular domain of c-MET. The radiolabeling of MET4 with ^125^I was accomplished by direct electrophilic radioiodination, as described in a previous study [45]. Nuclear images of c-MET-overexpressing mice tumor models injected with ^125^I-MET4 were qualitatively and quantitatively comparable to the best images published previously [43–45]. Moreover, biodistribution analyses revealed that MET4 is a durable antibody for in vivo applications, with an unusually long biological half-life of about 150 h (6.25 days) when compared to other full-length antibodies, which have a half-life of about 2–4 days, and therefore MET4 might be a candidate agent for radioimmunotherapy.

It is widely accepted that compounds labeled with positron-emitting radionuclides for PET imaging can produce images with higher resolution and which allow a more accurate quantitative analysis [48, 49]. Currently, only two positron-emitting radionuclides offer optimal tumor/nontumor ratios for intact mAbs (i.e., over 2–4 days): ^89^Zr (t_1/2_ = 78.4 h) and ^124^I (t_1/2_ = 100.3 h). Perk et al. initially reported the radiolabeling of DN30, a mAb reactive to c-MET labeled with ^89^Zr for assessing c-MET expression status in vivo by PET imaging [50]. The incorporation of ^89^Zr into DN30 was accomplished through a bi-functional metal chelating moiety conjugated to the mAb (N-sucDf-DN30). The radiolabeling achieved moderate yields of >70%, radiochemical purity of >95%, and specific radioactivities ranging from 54 to 70 MBq/mg. In biodistribution studies of xenografts in mice models, the uptake of ^89^Zr-N-sucDf-DN30 (^89^Zr-DN30) in FaDu (human head-and-neck cancer with low c-MET expression) tumors was significantly lower than that in GTL-16 (human gastric cancer with high c-MET expression) tumors, specifically, at 3 days p.i. 7.8 ± 1.2%ID/g was detected in FaDu tumors and 18.1 ± 4.5%ID/g in GTL-16 tumors. Consistent with these results, the radioactivity was more pronounced in the GTL-16 tumors when compared to FaDu tumors, and it is noteworthy that GTL-16 tumors as small as 11 mg were clearly visualized. Although sensitive for detection of small tumors, ^89^Zr-DN30 displayed relatively high uptake in the liver and spleen, partly due to residualization of ^89^Zr after catabolism of the conjugate in these organs. In summary, the DN30 monoclonal antibody accurately measures c-MET expression, as confirmed by IHC, and can be used for molecular imaging in vivo.

Onartuzumab, an experimental therapeutic 1-armed monoclonal antibody, was radiolabeled with ^76^Br or ^89^Zr and tested as a targeted molecular imaging agent in c-MET expressing cell lines and xenografts in mice models by Jagoda et al. in 2012 [51]. ^89^Zr-desferrioxamine (df)-onartuzumab was synthesized using a df-conjugate, with yields of >90% and specific radioactivities ranging from 0.037 to 0.185 MBq/μg. ^76^Br-onartuzumab was radiolabeled directly, with low yields of >45% and specific radioactivities of 0.118 ± 0.067 MBq/μg with high specific binding affinity for c-MET. In biodistribution studies, uptake in MKN-45 (human gastric carcinoma with high c-MET expression) tumors of ^76^Br-onartuzumab was about 2-fold higher than in U87-MG tumors (moderate level c-MET expression). However for ^89^Zr- df-onartuzumab, the uptake in the U87-MG tumors was 3-fold lower than that in the MKN-45 tumors at 5 days p.i., consistent with c-MET expression. Both ^76^Br-onartuzumab and ^89^Zr-df-onartuzumab had a similar uptake in non-target tissues in all tumor models. In blocking studies for MKN-45 xenografts, tumor uptake of ^89^Zr- df-onartuzumab decreased approximately 3.8-fold by plus of 10 nmol of unlabeled onartuzumab, suggesting that competitive blocking by unlabeled onartuzumab occurred in the tumor. In summary, ^76^Br-onartuzumab showed lower retention in the tumors when compared to ^89^Zr-df-onartuzumab, with clearance similar to the radioactivity in blood. For micro-PET imaging in MKN-45 xenografts, tumors injected with ^76^Br-onartuzumab could be detected as early as 18 h p.i., with optimal visualization at 24 h p.i. However, although tumors injected with ^89^Zr-df-onartuzumab could also be visualized at 18 h p.i., the image quality continued to improve over the 5-day period. ^89^Zr-df-onartuzumab accumulation in the normal tissues—blood, heart, lungs, gastrointestinal tract, and muscle—decreased slowly, with reduction of <50% from 18 h to 5 days, and in contrast, liver and kidney uptake increased over time, indicating hepatobiliary and renal clearance for ^89^Zr-df-onartuzumab. In addition, high bone uptake (up to 7%ID/g) at 5 days p.i. was observed, due to free ^89^Zr from the chelated tracer.

In 2014, Li et al. radiolabeled fully human single-chain variable fragments-cys-diabodies (scFv-cysdimmers, H2) with ^89^Zr for immuno-PET studies in xenografts in mice models expressing c-MET [52]. The ^89^Zr-labeled H2 cys-diabody (^89^Zr-DFO-H2 cys-diabody) was site-specifically conjugated with maleimide-DFO, and prepared with labeling efficiency of 49%, radiochemical purity of 95%, and specific activity of 0.13 MBq/μg. The ^89^Zr-DFO-H2 cys-diabody showed a specific uptake in Hcc827-GR6 tumors (gefitinib-resistant human non-small-cell lung cancer (NSCLC) with high c-MET expression) that was higher than the uptake in Hcc827 (human NSCLC with low c-MET expression) tumors in PET scans at both 4 and 20 h p.i.; high-contrast images were obtained as early as 4 h p.i. In ex vivo biodistribution assays, at 4 h after injection, Hcc827-GR6 tumors had higher uptake levels at 1.8 ± 0.2%ID/g when compared to the negative control Hcc827 tumors at 0.65 ± 0.15%ID/g. High kidney uptakes observed in both biodistribution and PET images were induced by clearance of these low molecular weight cys-diabody proteins. The specificity of anti-c-MET targeting by the H2 minibody (antibody fragment reformatted from H2 but with larger molecular weight) was also assessed in immuno-PET studies. Biodistribution analyses revealed higher specific uptake levels in Hcc827-GR6 tumors than other groups. However, the image contrast was not improved due to higher background signal in the blood (Hcc827-GR6 tumor: blood = 4.2 ± 0.5) resulting from the longer persistence of the minibody in the serum. Overall, the H2 cys-diabody is the preferred choice for rapid immuno-PET applications because it has the unique advantage of cross-reacting with mouse c-MET, thus allowing more representative imaging studies in the presence of endogenous c-MET expression in normal mouse tissues.

According to results of above studies, monoclonal antibodies or scFv specific to c-MET radiolabeled with the long half-life period positron-emitting radionuclide ^89^Zr could precisely detect c-MET expression in vitro and in vivo in mice tumor model, and may prove to be an effective immuno-PET agent in humans.

In addition to antibody-based radiotracers, antibody-based fluorescent tracers have also been used for c-MET-targeted molecular imaging in vivo to assess c-MET expression levels. In 2010, Lu et al. synthesized QD (quantum dot)-labeled anti-c-MET scFv (designated Ms20-QD) and tested its efficacy as an imaging agent in c-MET-expressing tumors [53]. The Ms20-QD synthesis was completed by site-directed conjugation of Ms20 cys to maleimide-activated quantum dots through its carboxy-terminal cysteine. In in vivo assays, xenografts in mice models derived from the human NSCLC cell line H1993, which has high expression levels of c-MET, were injected with Ms20-QD or non-conjugated QD and then imaged using a Xenogen IVIS 200 imaging system. At 6 h p.i., near-infrared (NIR) fluorescence signal intensity in the tumor tissues of Ms20-QD-treated mice was approximately 3.6-fold higher than in QD-treated mice. Biodistribution at 24 h p.i. revealed that Ms20-QD selectively accumulated in the H1993 xenograft tumor tissues with 4.3-fold more than QD alone.

Towner and his group made important breakthroughs in the domain of c-MET-targeted MRI, which offers better reproducibility without any radiation damage. In 2008, the team successfully visualized overexpressed c-MET in C6 (rat glioma) rat models in vivo by MRI based on a targeting contrast agent, anti-c-MET-Gd-DTPA-albumin, an anti-c-MET antibody linked to biotinylated Gd-DTPA-albumin [54]. T2-weighted images provided anatomical detail on tumor location, and T1-weighted images provided MR signal information of the Gd-based probe in glioma versus normal brain tissues. In contrast to MRI images collected before target agent administration, images taken at 3 h after injection of the anti-c-MET-Gd-DTPA-albumin showed that there was higher uptake in C6 tumor regions that persisted at least 24 h p.i. However, no significant temporal change in the MR signal was detected in the normal contralateral tissues. In addition, there were minimal changes in MR signal intensity and in T1 values in the C6 tumor region after administration of control probes, namely, non-Ab albumin-Gd-DTPA and a normal rat IgG-Gd-DTPA. Notably, a significant increase in MRI signal intensity in the outer regions of the glioma correlated with the vascular nature of the tumor, as described previously [55].

In another study [56], Towner et al. synthesized a molecular-targeted MRI contrast agent that could distinctively decrease MRI signal intensity and regional T2 values, by coupling SPIO (super paramagnetic iron oxide)-streptavidin to an anti-cMET antibody. SPIO-anti-c-MET was injected into a choline-deficient l-amino acid (CDAA)-defined rat hepatocarcinogenesis model and, for the first time, the expression of c-MET was visualized in vivo. A decrease in T2-weighted MRI signal, as well as changes in T2 values, were detected within selected regions of the tumor (expressing high c-MET), while two negative control groups including choline-sufficient l-amino acid (CSAA)-defined rats (normal c-MET levels) administered with the SPIO-anti-c-MET or with SPIO alone (without anti-c-MET antibody) showed no substantial changes in both T2 values or MRI signal intensities.

In 2010, Towner and colleagues reported another novel MRI molecular imaging probe for the in vivo detection of c-MET overexpression in C6 rat glioma models [57]. This probe, which consisted of a magnetite-based dextran-coated nanoparticle backbone covalently bound to an anti-c-MET antibody, also distinctively detected decreased MRI signal intensity and T2 values in tumor regions, when compared to normal tissue. As expected, the non-immune non-specific normal rat IgG coupled to dextran-coated nanoparticles control did not caused obvious changes in C6 tumor regions.

Based on data from above studies, Towner et al. initially developed these MRI contrast agents targeted to c-MET, and further illustrated that these anti-c-MET antibody-based targeting agents were feasible for detection of c-MET overexpression in tumor in vivo with MRI by changes in signal intensity. Despite this very valuable preclinical attempt to visualize c-MET using MRI, some limitations need to be considered such as nephrogenic systemic fibrosis (NSF) associated with MRI contrast agent Gd, immunogenicity from intact antibody [58]. On the other hand, nanoprobes based on SPIO possess higher security, with minimal impact on cell viability and function, and low toxicity [59].

#### Molecular imaging agents based on peptides

Compared to antibody-based c-MET-targeted molecular imaging, with inherent long clearance half-life after injection and potential immunogenicity, smaller peptide-based molecular imaging agents, due to their low molecular weight, present some advantages such as favorable pharmacokinetic and tissue distribution patterns, higher permeability, lower toxicity, less immunogenicity, and easy accessibility for chemical modification [60]. In recent years, a number of c-MET-targeted molecular probes based on a c-MET binding peptide (cMBP) have been developed that allow the visualization of c-MET expression in vivo.

In 2007, Zhao et al. searched for a cMBP from a random peptide phage display library using a subtractive planning approach on intact cells [61], and performed with a series of in vitro assays including competition ELISA, FACS analysis, cell internalization, and cell proliferation inhibition. They examined the specificity and binding affinity of the novel peptide, designated Met-pep1. Met-pep1reacts with c-MET on the cell surface and competes with HGF binding to c-MET in a dose-dependent manner. The authors tested Met-pep1 labeled with^125^I as a diagnostic tracer in vivo in xenografts in mice models of human leiomyosarcoma derived from the cell line SK-LMS-1/HGF, which expresses c-MET/HGF. Tumor-associated radioactivity was detected by nuclear imaging as early as 1 h after injection of the tracer and remained visible up to 24 h p.i. Quantitative ROI analyses showed that tumor activity accounted for 4 to 5% of the estimated injected activity at 1 and 4 h p.i. but declined to 3% at 7 h p.i. However, tumor-associated radioactivity detected after injection of a control ^125^I-labeled non-avid peptide was significantly lower at all time points. These results demonstrated that Met-pep1 was specific to c-MET and might be a potential diagnostic agent for clinical applications. Activity was highest in the liver at all imaging time points, suggesting that the liver is major metabolic pathway for the tracer. Nevertheless, activity was also evident in the bladder at 4 and 7 h post-injection, whereas thyroid activity derived from deiodination of the tracer increased over time.

**Fig. 3:**
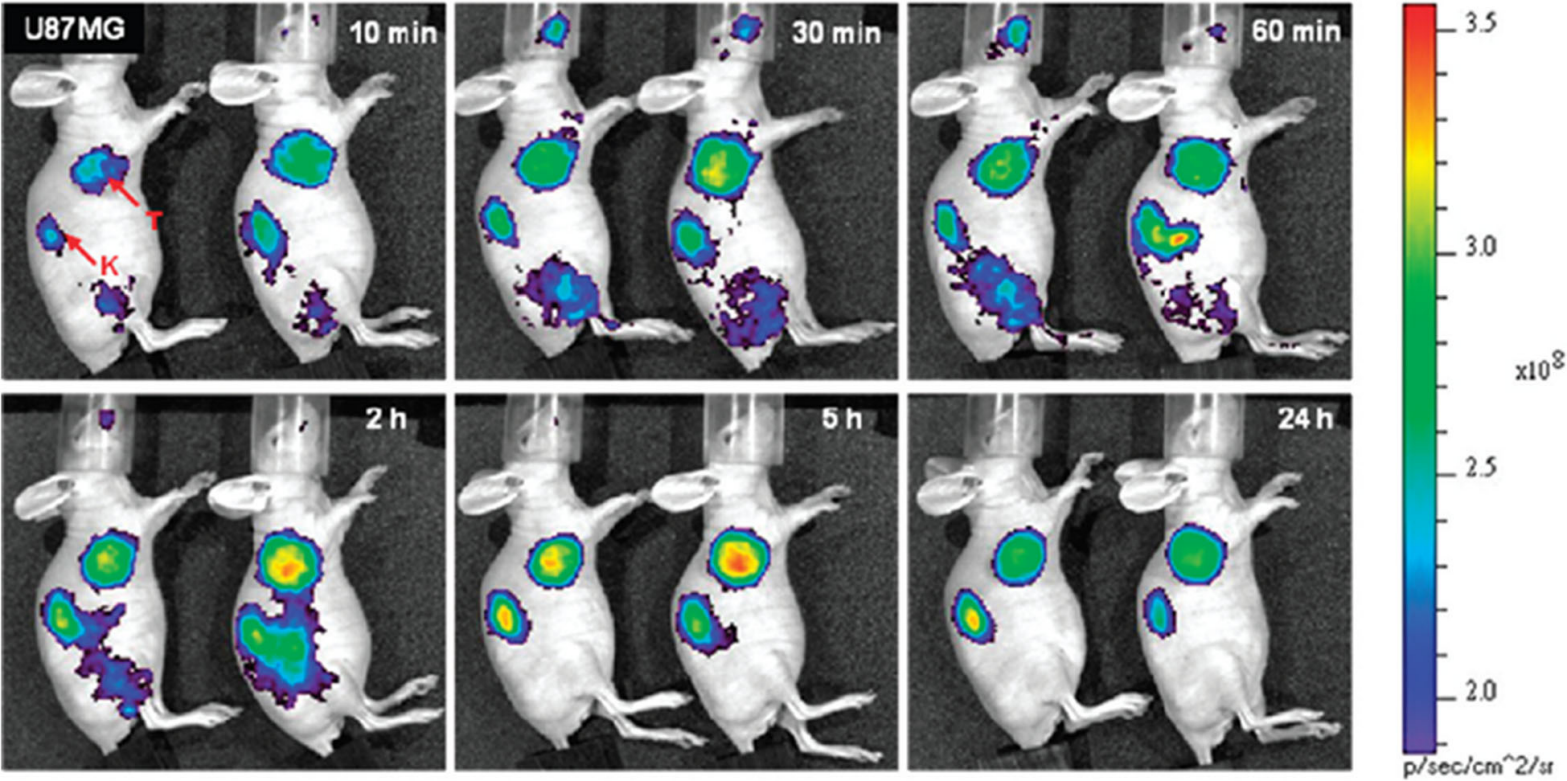
In vivo fluorescence imaging of U87-MG bearing xenografts after iv injection of cMBP-GGG-Cy5.5 (*left*) or cMBP-AOC-Cy5.5 (*right*), of which *red arrows* indicate the tumor (T) or kidney (K). With permission from reference [63]

In 2009, Kim et al. reported a new cMBP identified on a peptide phage display system and then radiolabeled with ^125^I directly, with a radiochemical purity of >90% [62]. As the biodistribution results showed that ^125^I-cMBP had low tumor-to-blood ratios (tumor/blood = 1.89 at 4 h p.i.), but a GGG (amino acid reducing hydrophilicity, tumor/blood = 2.94 at 4 h p.i.) or an AOC (aliphatic carbon increasing lipophilicity, tumor/blood = 1.78 at 4 h p.i.) were introduced as a linker to the structure of radiotracer enhance the tumor–cell binding affinity. Blocking assays showed that the specific binding to U87-MG cells was significantly blocked by unlabeled cMBP, cMBP-GGG, and cMBP-AOC, respectively, but not by the mismatched peptide. According to the biodistribution data, ^125^I-cMBP-GGG exhibited the highest tumor-to-blood ratios and tumor uptake over time. Consistent with these data, SPECT images of U87-MG xenografts were clearer in mice injected with ^125^I-cMBP-GGG. Unexpectedly, both cMBP-GGG and cMBP-AOC showed uniform high pancreatic uptake, which may be a result of peptide degradation, as there was very low c-MET expression in the pancreas compared to tumor tissue, as confirmed by RT-PCR. In addition, introduction of the linker did not affect specificity of the probe to c-MET, whereas a lower pancreatic uptake was observed. ^125^I-cMBP-GGG and ^125^I-cMBP-AOC could be cleared from body at 15 h p.i. mainly by renal metabolism. In the same year, based on the same peptide, Kim’s group reported two other c-MET-targeted probes labeled with cyanine dye 5.5 (Cy5.5), Cy5.5-cMBP-GGG, and Cy5.5-cMBP-AOC for optical imaging in athymic mice bearing U87-MG or Ramos (c-MET negative human B-lymphoma) xenografts [63]. The Cy5.5-conjugated peptides bound mainly to the cell surface with high binding affinities and could be blocked by free cMBP. U87-MG tumors were clearly visualized with both these fluorescent probes, but cMBP-AOC-Cy5.5 displayed higher tumor uptake and tumor-to-normal tissue ratios from 10 min to 24 h p.i. (Fig. 3). When cMBP-AOC-Cy5.5 was co-injected with cold cMBP in in vivo blocking assays, the tumoral uptake decreased up to approximately 35%. In a previous study, ^125^I-radiolabeled cMBP-GGG showed higher tumor uptake than cMBP-AOC [62]; however, tumor uptake was reversed by conjugation to Cy5.5. Indeed, while^125^I-radiolabeled cMBPs showed high pancreatic uptake, Cy5.5 conjugates were instead rapidly eliminated via the renal metabolic pathway. These differences in retention might be related to the size or properties of Cy5.5, for instance, lower pancreatic uptake could be caused by Cy5.5 hydrolysis. In conclusion, Kim et al. tested this cMBP radiolabeled with ^125^I or conjugated to Cy5.5 as a tracer with a different linker for visualization of c-MET. Results from these cMBP-based nuclear and optical images are in concordance, demonstrating that the cMBP-based tracer could accurately detect c-MET overexpression.

More recently, Burggraaf et al. reported a novel optically labeled c-MET-targeted molecular imaging probe, designated GE-137, which has been assessed in a preclinical study as well as in healthy volunteers [64]. GE-137 was prepared by conjugating a water-soluble 26–amino acid cyclic peptide (AH-111972) with high affinity for c-MET (*K*d = 2 nM) to a linker (GGGK) and subsequently to a modified Cy5 (Cy5**, λmax ex = 648 nm). Intravenous administration of GE-137 in mice bearing HT29 tumors (human colorectal cancer with positive c-MET expression) resulted in specific accumulation of the probe in c-MET expressing tumors, whereas Cy5**-labeled scrambled peptide as a control group was only visible in the kidneys. GE-137 has a dose-linear renal clearance, and the clearance rate was about 0.13 l/kg/h, and is comparable between dose groups. Plasma concentrations were 0.12–0.16 mg/L at 3 h after administration of 0.13 mg/kg. In addition, co-administration of GE-137 with an excess of unlabeled peptides significantly reduced tumor uptake. After being validated as safe and well tolerated in healthy volunteers, GE-137 was tested in cancer patients. GE-137 visualized by fluorescence colonoscopy could detect not only all neoplastic polyps that were visible with white light but also additional polyps with c-MET expression, as verified by immunohistochemical analyses. This landmark first-in-human pilot study conducted by Burggraaf et al. showed that molecular imaging using an intravenous fluorescent c-ME- targeted agent is a feasible and safe method for clinical applications.

Liu et al. [65] showed using a fluorescent imaging device developed in-house that GE-137 specifically accumulated in SKOv3 (high c-MET expression human oophoroma) xenografts in mice and peaked at 2–3 h after injection, and lasted for up to 8 h, whereas fluorescence intensity was comparatively lower in MUKA xenografts in mice (low c-MET expression human oophoroma). In addition, no accumulation of AH11444, a Cy5 derivative used as a negative control tracer, was observed in the tumor, further demonstrating the selectivity of GE-137 in tumor uptake. Strikingly, even submillimeter SKOv3 tumor deposits were observed subcutaneously in real time, revealing the high sensitivity of the fluorescence imaging device in detecting c-MET expressing tumors.

In summary, the novel fluorescent tracer based on c-MET-targeted peptide (GE-137) has successfully visualized expression of c-MET in preclinical study and was further used to detect colorectal carcinoma overexpressing c-MET in clinical setting. Furthermore, the peptide targeted against c-MET was also labeled with radionuclides (^18^F, ^99m^Tc) to detect expression of c-MET.

Based on these studies, in 2016, Arulappu et al. developed a radiolabeled analogue of GE-137, ^18^F-AH113804, synthesized with radiochemical purity of >90% and specific activity of 100 GBq/μmol [66]. After validating the high affinity and specific binding of ^18^F-AH113804 to c-MET (Kd ≈ 2nM) by ex vivo analyses and PET imaging, Arulappu and colleagues demonstrated that ^18^F-AH113804-based PET imaging could detect loco-regional recurrences after surgical excision of orthotopically implanted HCC1954 human basal-like breast cancer with high c-MET expression in mice models as early as 6 days postoperatively, while with CT scanning, the recurrences could only be visualized after at least 20 days. These results showed that ^18^F-AH113804-based PET imaging has great potential as a clinical screening tool for earlier loco-regional recurrences of human basal-like breast cancer. Due to smaller molecular weight (~3.2 kDa), 18 F-AH113804 not only permitted early imaging after tracer administration (1 h p.i.) but also cleared fast from plasma and non-target tissues (such as liver, lungs, and heart), allowing for high-contrast imaging after injection, and improving the sensitivity for tumor detection.

Remarkably, Jagoda and colleagues not only conducted leading work in the development of antibody-based c-MET-targeted molecular imaging but they also performed extensive research on molecular imaging based on cMBP [67, 68]. The team was able to modify a 26-amino acid cMBP AH-111972 at the C-terminal lysine with cPn216 chelating for ^99m^Tc, or with tetra SO_3_ for labeling with cy5** (653/675 nm). ^99m^Tc-cMBP was synthesized with radiochemical purity of >90% and specific activity of 430 Ci/mmol, and with high affinity binding (nM) to c-MET observed in vitro in MKN-45 (high c-MET expression). In biodistribution assays of MKN-45 xenografts in mice models, higher uptake of ^99m^Tc-cMBP was detected in MKN-45 xenografts than all organs except for the kidney. Blocking studies showed that tumor uptake decreased by 62% after coinjection of ^99m^Tc-cMBP with 24 nmol of unlabelled cMBP. In initial microSPECT imaging studies, the tumor could be visualized as early as 30 min p.i. and clearly at 60 min p.i., and the images remained relatively constant until 120 min p.i. but then the signal significantly decreased. Measurements of tumor vs background signal indicated that the optimal imaging time for ^99m^Tc-cMBP was 90 min p.i., and this time point was therefore adopted in subsequent longitudinal imaging studies for assessing the ability of ^99m^Tc-cMBP to monitor targeted therapy in MKN-45 xenografts with c-MET TKI PHA665752. ^99m^Tc-cMBP tumor uptake in the treated group, when compared to the vehicle group, was decreased by 43, 42, and 25% at days 8, 14, and 21, respectively. Notably, these results were comparable to cy5**-cMBP fluorescence imaging using the same treatment models with an optimal dose of 1 nmol and optimal imaging time of 60 min p.i.., and this was validated by histologic and immunohistochemical analyses. ^99m^Tc-cMBP and cy5**-cMBP imaging may therefore have clinical applications in diagnostic and therapeutic monitoring in the future.

#### Molecular imaging agents based on genetically encoded proteins

The strategies that whole body optical (e.g. fluorescence, bioluminescence) imaging based on genetically encoded proteins have been critically evaluated as a feasible molecular imaging modality in cells and transgenic animals, enabling noninvasive, longitudinal studies of dynamic biological processes in vivo [69, 70].

In 2011, a study by Zhang et al. reported a novel method for c-MET-targeted molecular imaging and therapy monitoring based on gene transfected mice models [71]. Plasmids carrying bioluminescent reporters for wild type c-MET (BMRwt) or c-METY530A (BMRmut) that turned OFF when c-MET was active/phosphorylated, and ON when c-MET was inhibited, were constructed and then stably transfected into human glioma cell lines D54 and U87-MG (both with c-MET expression) to assess their ability for detecting changes in c-MET expression. U87–BMRwt cells that were treated with increasing doses of the c-MET inhibitor SU11274 exhibited increased bioluminescence activity in a dose-dependent manner, peaking at 15 min before plateauing thereafter. In contrast, cells transfected with BMRmut, which are resistant to SU11274, had no significant change in bioluminescence activity in response to SU11274 treatment. In vivo assays in murine models using an IVIS imaging system showed that tumors in control antibody-treated animals had no significant increase in bioluminescence activity after SU11274 administration, while HGF neutralizing antibody-treated tumors had a 4- to 5-fold increase in bioluminescence activity at 3 h p.i. and sustained for 10 h.

In 2006, another method for c-MET-targeted molecular imaging based on genetic engineering was developed [72]. In this study, Moshitch-Moshkovitz et al. constructed a GFP (green fluorescent protein)-Met chimeric molecule that functioned similarly to wild type c-MET, and then generated GFP-c-MET transgenic mice. These mice ubiquitously expressed GFP–Met in specific epithelial and endothelial cells and displayed enhanced GFP–Met fluorescence in sebaceous glands with the fact that 32% of the males spontaneously developed adenomas, adenocarcinomas, and angiosarcomas in their lower abdominal sebaceous glands. Quantitative subcellular resolution intravital imaging revealed very high levels of GFP-c-MET in tumor lesions and in single isolated cells surrounding them, relative to normal sebaceous glands. These single cells with higher GFP-c-MET levels were correlated to early tumor aggressiveness and preceded the formation of local and distal metastases.

#### Molecular imaging agents based on anticalins

**Fig. 4:**
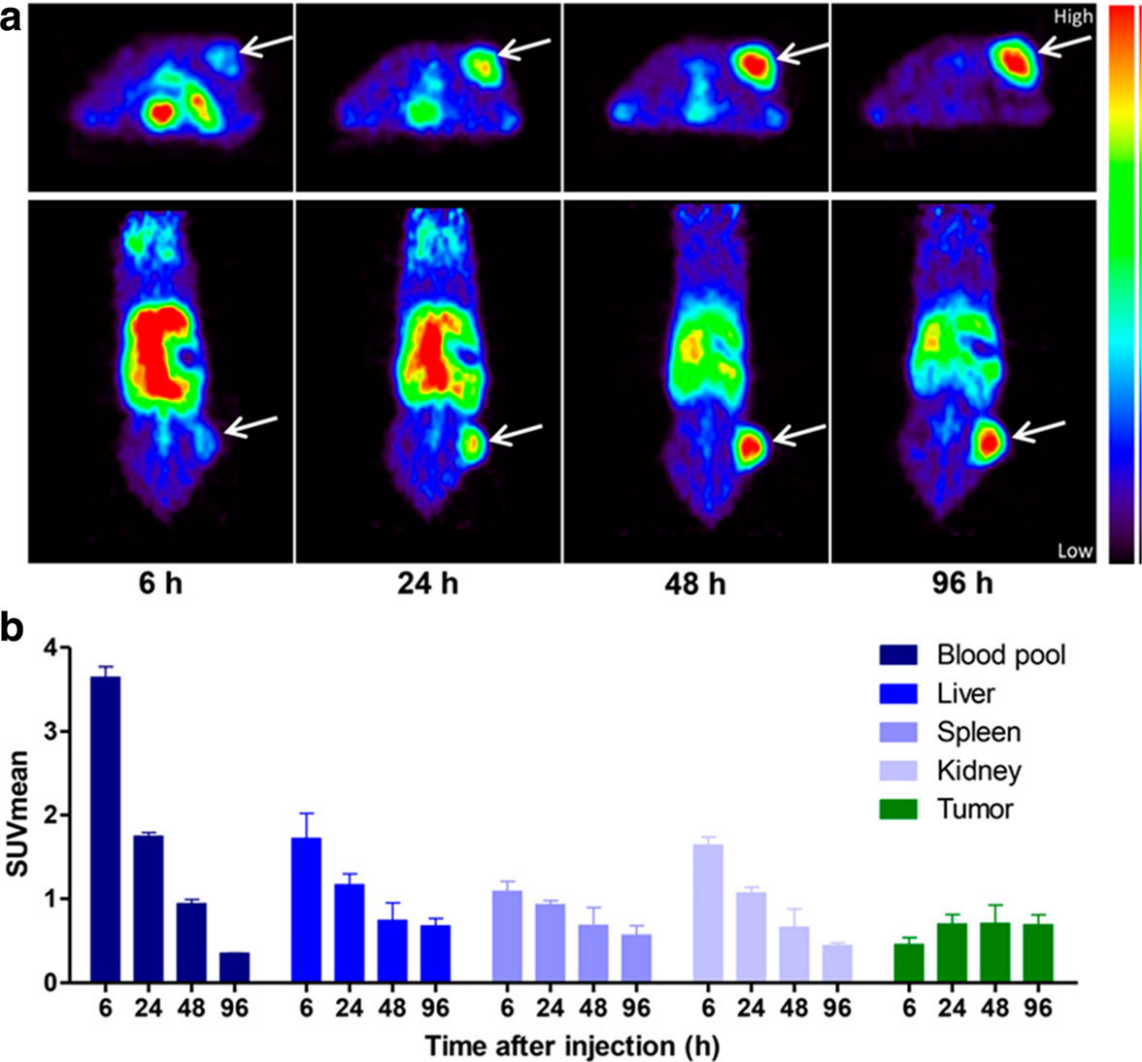
^89^Zr-PRS-110 micro-PET imaging of H441-bearing mice. **a** Transversal and coronal micro-PET images of the mice are shown at different timepoint post-injection of tracer. **b** Micro-PET data quantification expressed as mean standardized uptake value (SUVmean) was performed for normal organ and tumor in all mice. With permission from reference [74]

Anticalins are a novel class of protein-based biopharmaceuticals that due to their small size (17 kDa) may have more favorable tumor uptake and penetration when compared with the much larger IgGs (150 kDa), and therefore display highly desirable attributes as molecular imaging agents. Terwisscha van Scheltinga et al. engineered PRS-110, an anticalin with monovalent specificity for c-MET (binding affinity of 0.6 nmol/L), which is site-specifically conjugated to a branched 40-kDa polyethyleneglycol (PEG) (2 · 20 kDa PEG) moiety for half-life prolongation [73, 74]. A phase I trial in cancer patients supports the use of the anticalin drug platform for broad therapeutic and diagnostic applications [75]. To visualize in vivo c-MET expression and study the organ distribution of this anticalin, Terwisscha van Scheltinga and colleagues generated ^89^Zr-PRS-110 with a specific activity of 100 MBq/mg and radiochemical purity of >95% and assessed its specific uptake in different human tumor xenograft models. Ex vivo assays showed that increasing doses of unlabeled PRS-110 led to tumor saturation and to specific blockade of ^89^Zr-PRS-110 uptake. PET imaging performed in H441 bearing mice (human NSCLC with high c-MET expression) with a suitable concentration of the tracer revealed that tumor uptake peaked at 48 h p.i. (Fig. 4) and showed a significantly higher uptake of ^89^Zr-PRS-110 when compared to ^89^Zr-Tlc-PEG (non-c-MET binding), in line with the ex vivo biodistribution data (5.9 vs. 3.9%ID/g). In A2780 bearing mice (human ovarian cancer with negative c-MET expression), both ^89^Zr-PRS-110 and ^89^Zr-Tlc-PEG were aggregated up to a similar extent (1.7 and 2.5%ID/g respectively), with lower uptake than H441 tumors. Overall, there was a significant correlation between ex vivo biodistribution and small-animal PET data quantification (*R* ^2^ = 0.812; *P* < 0.05). In addition, uptake of ^89^Zr-PRS-110 and ^89^Zr-Tlc-PEG in various organs was comparable, except for the lung. Lung uptake of ^89^Zr-PRS-110 in mice bearing H441 tumors occurred neither in A2780 tumors with ^89^Zr-PRS-110 nor in any of the two xenografts with the negative control ^89^Zr-Tlc-PEG. The decreased uptake in the absence of c-MET and for the non-binding control demonstrates a c-MET-associated mechanism of uptake in the lung (possibly metastasis), which needs further investigation.

Anticalin is a novel biopharmaceutical with good drug properties that may be applied extensively in the clinic, as anticalin-based molecular imaging could not only be useful for the diagnosis at the molecule level but also enable real-time monitoring of anticalin metabolic pathways (e.g., PRS-110) and pharmacokinetics, thus having a dual role in molecular imaging and drug development.

#### Molecular imaging agents based on small molecules

The binding sites for the imaging tracers described above are all located on the extracellular domain of the c-MET receptor; however, the initial molecular event that leads to malignant development is the abnormal activation of the receptor’s tyrosine kinases, which are located in the intracellular domain of c-MET, and which will ultimately trigger downstream signaling pathways. Thus, molecular imaging based on small molecule probes with intracellular binding site can provide more roundly information about the c-MET activation status. Small molecule imaging probes, with their low molecular weight and size, are rapidly cleared and have higher permeability, and in particular, those based on c-MET-TKI have attractive properties, such as being non-toxic and easily accessible for clinical translation. However, to date, little progresses have been made in the development of small molecule imaging agents.

The first reported study by Wu et al. in 2010 [76] described the small molecule c-MET-TKI SU11274, a reversible c-MET inhibitor that was designed and synthesized as a labeling precursor and was subsequently methylated with ^11^C-methyl iodide to produce ^11^C-SU11274 with radiochemical purity of ≥98% and a specific activity of 0.3–0.5 Ci/mmol. ^11^C-SU11274 has the same structure as SU11274, and therefore, its binding affinity and specificity was preserved. Then the authors tested ^11^C-SU11274 as a micro-PET imaging agent for c-MET expression in vivo. They showed that tumor uptake of ^11^C-SU11274 in c-MET-positive H1975 bearing mice (human NSCLC) was significantly higher than that in c-MET-negative H520 (human NSCLC) xenografts. Specifically, tumor uptake of ^11^C-SU11274 in H1975 peaked up at 80 min p.i. and was always higher than that in H520. These results not only confirmed the feasibility of using ^11^C-SU11274 as a PET tracer for c-MET activation but also revealed the importance of the tracer to study pharmacokinetics of SU11274.

The slow progress in the development of small molecule imaging agents based on c-MET-TKI could mainly be attributed to challenges in the modification and synthesis of the probes. Indeed, the synthesis of the labeling precursor, which is modified from the small molecule c-MET-TKI, should produce a structure that is same or similar to the parent drug after labeling, in order to preserve the specificity to the target. Moreover, the labeling reaction requires a very complex and precise technique that is crucial to synthesize stable and pure imaging tracers.

## Conclusions

In this review, we have provided a brief historical overview of the development of c-MET-targeted imaging agents, from mAbs to small molecules. Evidently, each substrate, whether it is an endogenous ligand, antibody, peptide, or small molecule, has unique advantages and limitations. While radiolabeled mAbs such as ^89^Zr- df-onartuzumab have shown promise in preclinical studies, smaller structures like anticalin and peptides also demonstrated similarly desirable in vivo properties, making them compatible for radiolabeling with the more popular radionuclides ^18^F or ^99m^Tc, which have shorter half-lives and have been extensively used in clinical applications.

In general, various types of molecular probe have distinctive advantages and disadvantages. For example, antibody-based probes have high binding affinity but with long metabolic cycle and immunogenicity. And small molecule probes have advantages in size, pharmacokinetics, and security, but are inadequate for delayed imaging after injection, due to fast clearance. Regarding the imaging technique, nuclear imaging (e.g., PET, SPECT) are useful for imaging whole body and deep viscera, whereas optical imaging (e.g., fluorescence, bioluminescence) is mainly applied in detecting superficial organs and endoscopic procedures, due to weak penetrability of fluorescence signal.

In our opinion, after clinical application of GE-137 with encouraging results for detection of malignant polyps, the field of c-MET-targeted molecular imaging will hopefully become established in the clinic with many expected research results in preclinical studies. Other strategies which might be relevant for translation into the clinic are antibody (fragment) tracers, as companion diagnostics for their parental antibody therapies [77]. Although studies for c-MET-targeted molecular imaging have made many important advances, most of imaging agents specifically target to extracellular area of c-MET, so it can only reflect overexpression of c-MET. Nevertheless, imaging agents based on small molecule c-MET-TKI possess intracellular binding site as well as many advantages, thus should be paid more attention.

c-MET-targeted molecular imaging makes it possible to visualize the abnormal alteration of c-MET expression in vivo and in real time, with the potential applications such as patient screening for c-MET-targeted therapeutics, monitoring therapeutic effects, evaluating prognosis, and analysis for resistance mechanism. Although the optimization of c-MET-targeted molecular imaging for clinical purposes still faces many challenges, the recent advances in the field, together with its potential clinical benefits, are highly encouraging for future research.

## Abbreviations

%EIA: Percentage of estimated injected activity
BOLD: Blood oxygenation level dependent
CDAA: Choline-deficient l-amino acid
cMBP: c-MET binding peptide
c-MET: Mesenchymal–epithelial transition factor
CSAA: Choline-sufficient, l-amino acid
EGFR: Epidermal growth factor receptor
ELISA: Enzyme-linked immuno sorbent assay
Fab: Antigen-binding fragment
FACS: Fluorescence-activated cell sorter
FISH: Fluorescent in situ hybridization
GFP: Green fluorescent protein
HER2: Human epidermalgrowth factor receptor-2
HER3: Human epidermalgrowth factor receptor-3
HGF: Hepatocyte growth factor
IgG: Immunoglobulin G
mAbs: Monoclonal antibodies
MAPK: Mitogen-activated protein kinase
MRI: Magnetic resonance imaging
NSCLC: Non-small-cell lung cancer
NSF: Nephrogenic systemic fibrosis
PcAb: Polyclonal antibody
PDGFR: Platelet-derived growth factor receptor
PEG: Polyethyleneglycol
PET: Positron emission tomography
QD: Quantum dot
RAS: Rat sarcoma
ROI: Region of interest
RON: Recepteur d’origine nantais
RTK: Receptor tyrosine kinase
RT-PCR: Reverse transcription-polymerase chain reaction
SPECT: Single photon emission computed tomography
SPIO: Super paramagnetic iron oxide
TKI: Tyrosine kinase inhibitor
